# Dr. Edward H. Clarke, of Boston, Mass.

**Published:** 1878-01

**Authors:** 


Dr. Edward H. Clarke, of Boston, Mass., died Nov. 30th, of cancer of the rectum, in the 57th year of his age. He graduated at Harvard in 1841; at the medical department of University of

Pennsylvania, in 1846. In 1850 he and others started the Boylston Medical School, and in 1855 he was appointed Professor of Materia Medica in the Harvard Medical School, which position he held until 1872. He was an interesting and popular writer, but he will be principally known by his essays on Sex in Education, and his paper on Practical Medicine in the Century of American Medicine. He was a very successful lecturer, and had the reputation of making "materia medica interesting."—*The Med. Record.*

